# Silver-Induced Contact Dermatitis From Electrocardiography Monitoring Electrodes: A Case Report

**DOI:** 10.7759/cureus.110299

**Published:** 2026-06-05

**Authors:** Ali K Hasan, Qasim M Shamtoot, Ali M Juma, Maawa M Juma, Mariam AlTurani, Zahra H Altaif, Fatima M Amini

**Affiliations:** 1 General Practice, Salmaniya Medical Complex, Manama, BHR; 2 Internal Medicine, Salmaniya Medical Complex, Manama, BHR; 3 Medicine and Surgery, Salmaniya Medical Complex, Manama, BHR; 4 Emergency Department, Salmaniya Medical Complex, Manama, BHR

**Keywords:** adhesive allergy, allergic contact dermatitis (acd), ecg electrodes, irritant contact dermatitis, silver allergy

## Abstract

Twelve-lead electrocardiography is among the most commonly performed and essential investigations in hospitals worldwide. This paper describes the development of suspected silver-induced contact dermatitis in a 57-year-old patient requiring continuous cardiac monitoring in the cardiac intensive care unit (CCU). The patient developed multiple, well-demarcated erythematous annular lesions over the chest following exposure to silver-containing electrocardiographic electrodes.

The patient received multidisciplinary care addressing his heart failure, obstructive uropathy, and contact dermatitis before being transferred to a better-equipped national cardiac center for optimal management of his acute cardiac illness. Dermatological manifestations associated with silver-containing electrodes are rarely reported in the literature, further emphasizing the importance of thorough history taking and routine physical examination in daily clinical practice. This case also highlights the need for physicians to maintain a high index of suspicion for contact dermatitis related to medical devices, enabling prompt recognition of complications and the formulation of individualized management plans tailored to each patient's clinical needs. As confirmatory patch testing was not performed, a definitive diagnosis of silver allergy could not be established.

## Introduction

Electrocardiography (ECG), invented in 1901 by William Einthoven using string galvanometry [1], is one of the most important inventions in modern medicine. Since 1901, technological advancements have enabled ECGs to become non-invasive, inexpensive, and widely available, making them the most common go-to investigation worldwide for evaluating cardiac pathologies [2].

To obtain an adequate ECG, clinicians place 12 electrodes at different sites on the patient’s body, with 4 limb and 6 chest leads. These electrodes commonly contain Silver (Ag/AgCl), surrounded by conductive gel and adhesive material [3]. Silver is generally considered hypoallergenic; however, like any other chemical, it can induce dermal hypersensitivity reactions, causing itching, redness, rash, and swelling [4].

Contact dermatitis secondary to chemical exposure is classified as allergic contact dermatitis (ACD) and irritant contact dermatitis (ICD). Differentiating ACD from ICD can be challenging because both conditions commonly present with erythema, pruritus, and localized inflammation at the site of exposure. ACD is a type IV delayed hypersensitivity reaction that develops after prior sensitization to a specific allergen and may spread beyond the area of direct contact. In contrast, ICD results from direct chemical or physical damage to the skin barrier and is generally confined to the exposed area. Careful assessment of the patient's history, timing of symptom onset in relation to exposure, lesion characteristics, and recurrence following re-exposure can aid in distinguishing between the two conditions. When the diagnosis remains uncertain, patch testing serves as the gold standard for confirming ACD and identifying the responsible allergen [3,4].

This paper describes a rare and previously unreported presentation and course of contact dermatitis secondary to continuous cardiac monitoring with silver ECG electrodes in a patient admitted with cardiac pathology.

## Case presentation

A 57-year-old male on warfarin, bisoprolol, and omeprazole, and known to have morbid obesity, hypertension, diabetes mellitus, and fast atrial fibrillation, presented to the emergency department after being referred by a private cardiologist. He had a one-week history of worsening shortness of breath, fatigue, bilateral lower limb swelling, orthopnea, and a productive cough of yellowish sputum. He had no known drug allergy. The patient denied any history of fever, sore throat, hemoptysis, chest pain, paroxysmal nocturnal dyspnoea, abdominal pain, rashes, weight changes, dizziness, nausea, vomiting, or any other symptom.

On examination in the emergency department, the patient was alert, conscious, and fully oriented. He was seated on the bed in marked respiratory distress. He was afebrile, tachycardic (130-170 beats/min), hypertensive (158/88 mmHg), and tachypneic (22 breaths/min), while maintaining oxygen saturation on room air. Chest examination revealed bilateral crepitations with bronchial breathing and a left-sided wheeze. Cardiac examination demonstrated a grade 2 systolic murmur at the apex and a grade 2 diastolic murmur in the aortic region. Examination of the lower limbs showed bilateral pitting edema, extending from the malleoli to the proximal tibiae below the knees.

The patient was referred to the cardiology department and admitted to the critical cardiac care unit for further evaluation and monitoring as a suspected case of decompensated heart failure. A transesophageal echocardiogram was performed, as the previously done transthoracic echocardiogram contained vague findings because of the patient's morbid obesity. The transesophageal echocardiogram showed severe mitral valve prolapse with regurgitation, a patent foramen ovale, mild tricuspid regurgitation, and a preserved left ventricular ejection fraction (>60%). In line with hospital guidelines, the patient was considered a candidate for mitral valve repair or replacement. However, since the main governmental hospital had limited resources for proper valve replacement procedures, a request for an official transfer to a more specialized cardiac centre was made.

While awaiting transfer to the National Cardiac Centre, the patient’s renal function began to deteriorate, prompting further evaluation with a renal ultrasound and a non-contrast CT scan. Imaging revealed large obstructive ureteric stones causing severe hydronephrosis. The urology team was consulted, and the patient subsequently underwent percutaneous nephrostomy followed by double J stent insertion.

**Figure 1:**
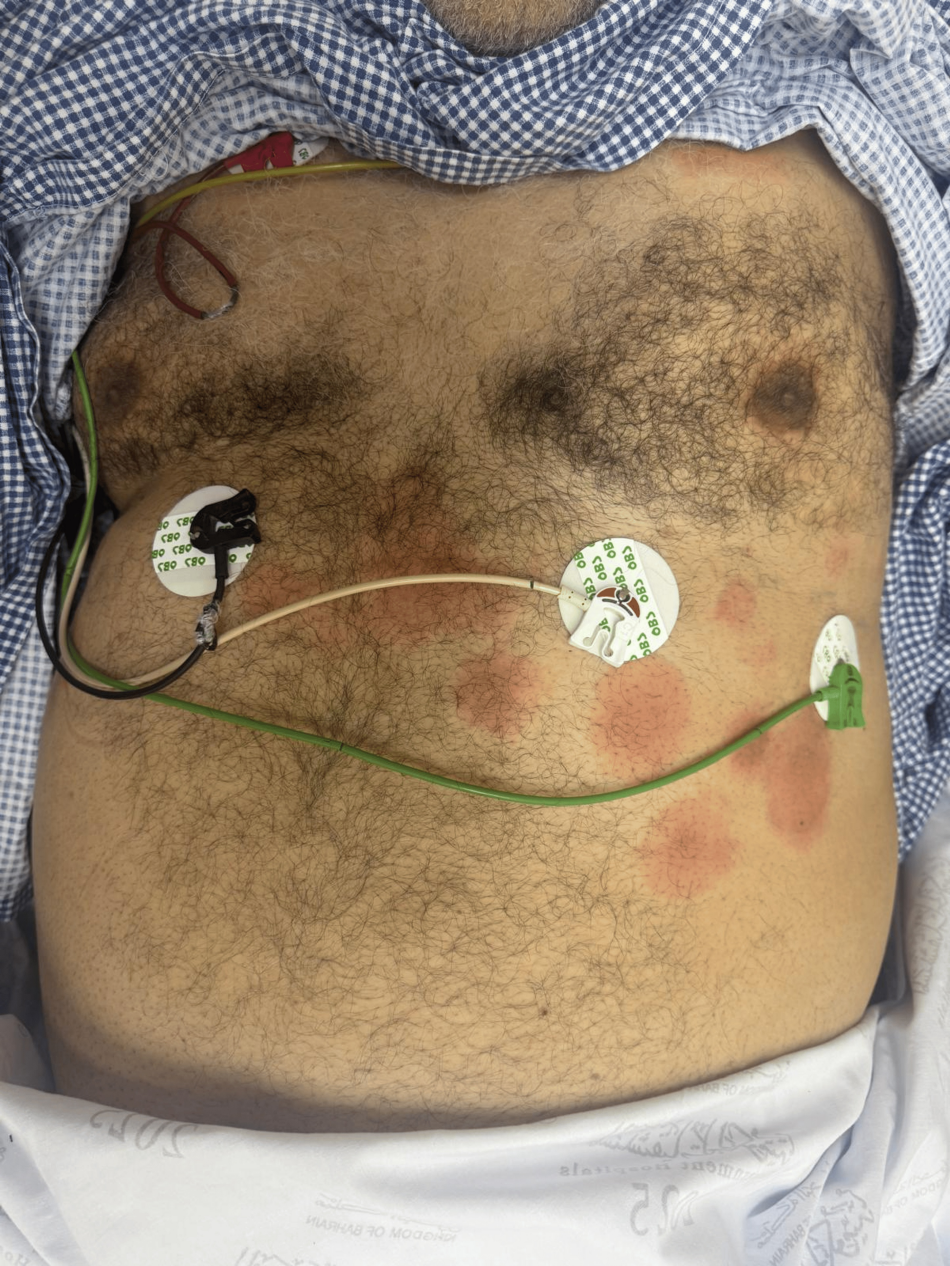
Day 1 of admission: Development of circular erythematous skin lesions, taking the shape of the ECG electrodes

**Figure 2:**
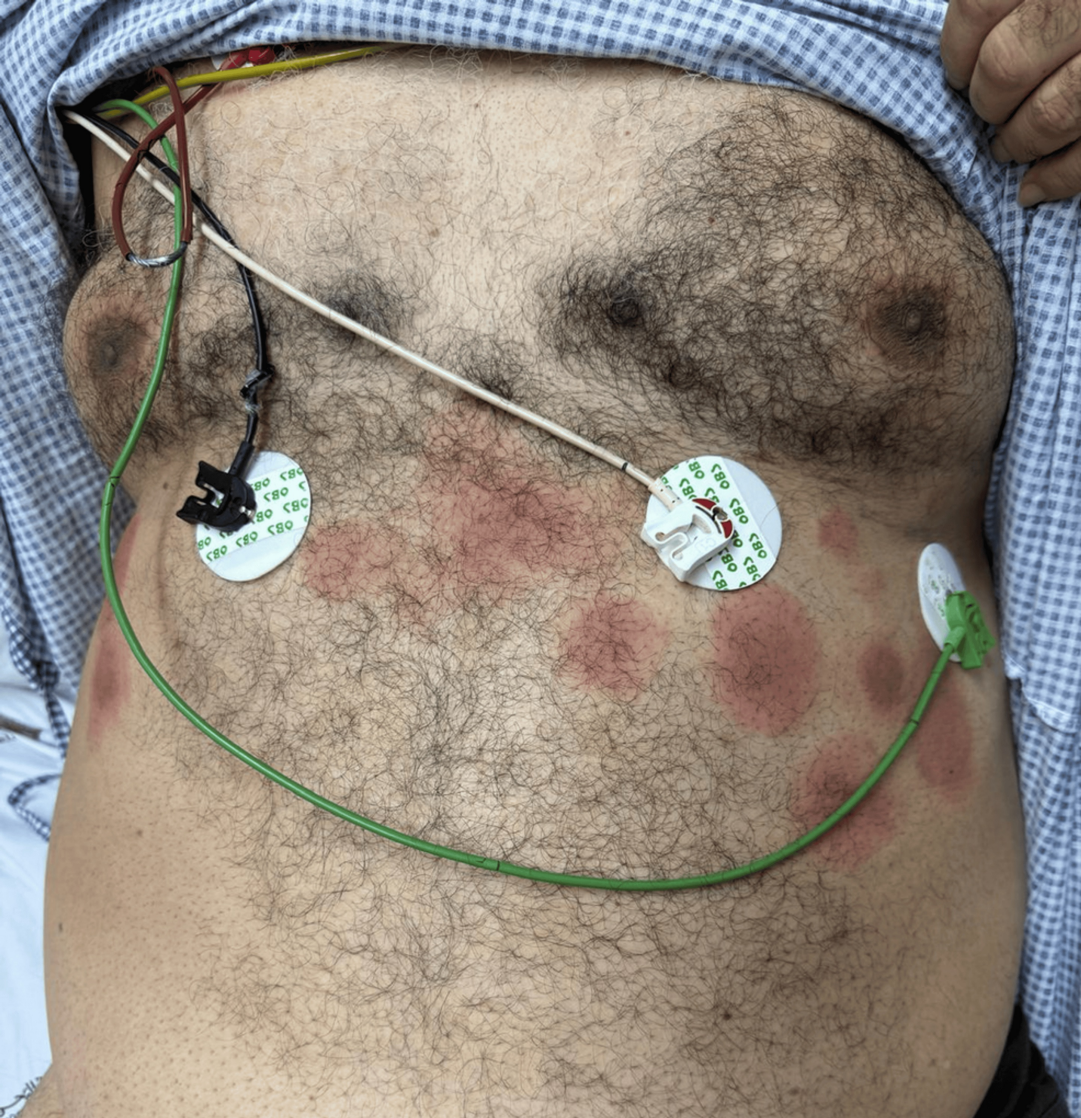
Day 4 of admission: Lesion progression into a darker red color over ECG electrode placement areas; the nurses tried to avoid placing electrodes over the affected skin

**Figure 3:**
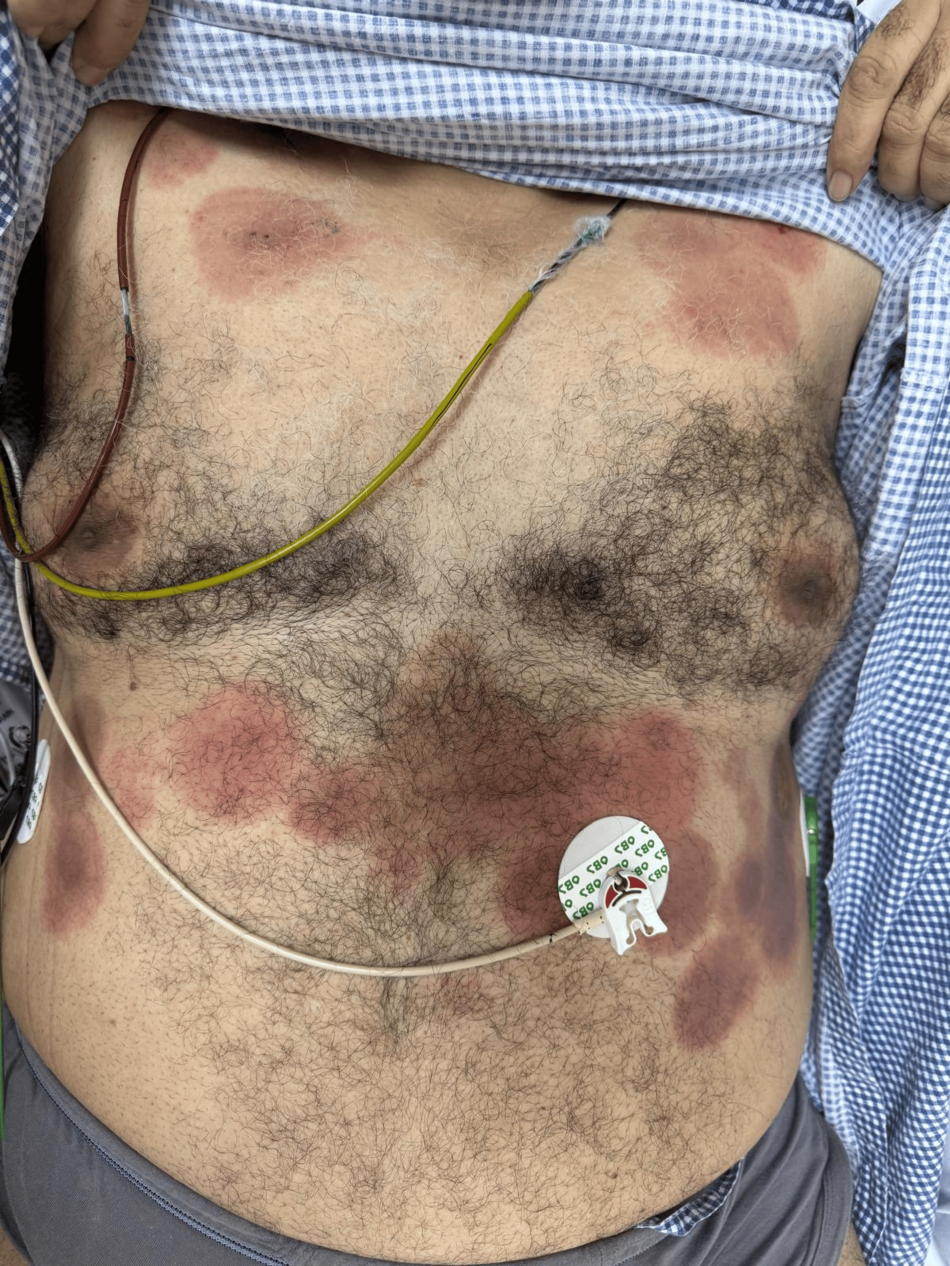
Day 11 of admission: Lesions increased in intensity with a widespread pattern and purplish change in color

Following the procedures, the patient developed numerous annular erythematous itchy patches varying in size at the corresponding anatomical sites (anterior trunk and the limbs) where silver chloride ECG electrodes remained in place for around 11 days (Figures 1-3), with no other bodily area affected. According to the patient, this was the first time that this happened, although he reported a similar rash whenever he wore silver jewellery. Other brands or types of electrodes were not available at the time to compare the effect of their components on the patient. The dermatology department was consulted, and they concluded that the patient had developed silver-induced contact dermatitis. Since the ECG monitoring leads could not be removed owing to the patient’s condition, they advised treating symptomatically with topical fucidic acid + betamethasone twice daily for three days, then as needed.

Despite treatment, the patient still complained of severe itchiness over the areas of the rash. Dermatology was consulted again, and they advised switching to Mometasone ointment twice daily. After two days, the rashes progressed and enlarged in the defined area with surrounding erythema and some bleeding because of the itching. Dermatology advised continuing the Mometasone regimen, adding an antihistamine like Cetirizine, and using Fluticasone nasal spray on areas of ECG placement 20 minutes prior to their attachment. This off-label strategy aims to reduce cutaneous inflammation through local corticosteroid effects and has been reported in isolated cases of medical device-associated contact dermatitis. However, the supporting evidence is limited primarily to case reports and expert opinion, and no standardized guideline currently recommends its routine use for ECG electrode-associated dermatitis [5]. Patch testing was not done because of the clinical condition of the patient.

The patient was then referred to the National Cardiac Centre for follow-up pertaining to his surgery, and it was reported that after the ECG electrodes were no longer used after the surgery, he improved gradually.

## Discussion

Most diagnostic procedures, along with medical and surgical treatments, can predispose to localized and, less commonly, systemic contact dermatitis through multiple routes of exposure, including topical, oral/mucosal, intravenous, intramuscular, inhalational, and via implantable devices. Early recognition of the signs and symptoms of contact dermatitis enables prompt identification and avoidance of the causative allergen or irritant, leading to significantly improved patient outcomes both during hospitalization and after discharge [4].

Silver itself exhibits hypoallergenic, antibacterial, anti-inflammatory, and antioxidant properties, making it very versatile in clinical environments. In the literature, silver is rarely associated with adverse skin reactions; when it is, silver-induced contact dermatitis is usually the sequela [6]. This relates to T-cell-mediated hypersensitivity reactions to metals in general, specifically silver. Silver ions penetrate the epidermis, where they bind to skin proteins, forming haptens that activate antigen-presenting cells and trigger a T-cell-mediated immune response [4]. This mechanism aligns with the delayed onset of ACD symptoms, typically occurring days after exposure.

Although other components of the ECG electrodes may have contributed to the patient's reaction and cannot be completely excluded as potential allergens, the patient's history of developing similar symptoms following exposure to silver-containing items, such as silver watches and rings, makes silver the most likely culprit. Nevertheless, in the absence of patch testing, it remains difficult to definitively identify silver as the sole causative allergen or to exclude the possibility of sensitization to other electrode constituents.

Despite the limited number of reported presentations of suspected silver-induced allergic contact dermatitis, clinicians should remain vigilant and carefully monitor localized inflammatory reactions following hospital-based interventions. Early recognition and appropriate management may reduce morbidity and facilitate continuation of essential medical care. While the clinical findings and timing of symptom onset were highly suggestive of contact dermatitis secondary to silver-containing electrodes, confirmatory patch testing was not performed because it was unavailable at the treating institution and prohibitively expensive in the local private healthcare sector. Therefore, the diagnosis remains presumptive rather than definitively confirmed.

Management of contact dermatitis requires patient cooperation and careful consideration of the risks and benefits related to continued allergen exposure and necessary medical interventions. The cornerstone and first-line treatment is the removal of the offending agent and avoidance of further exposure to the dermatitis-inducing material [7]. In this case, due to the patient’s critical cardiac condition, eliminating exposure to the ECG electrodes was unsafe and could not be done; hence, the progression of the dermatological manifestation and worsening of the patient’s skin condition.

In addition to allergen removal, symptomatic management of the rash is an important aspect of treatment. Acute dermatitis generally responds well to hydrophilic topical preparations, whereas chronic dermatitis is more effectively managed with water-in-oil-based ointments. If emollient therapy alone is insufficient, escalation to topical corticosteroids may be considered, particularly with class II and III corticosteroids in acute cases. Other therapeutic options include ultraviolet therapy for chronic dermatitis and coal tar preparations, which exhibit antiphlogistic and antiproliferative effects [7].

Beyond topical therapy, systemic treatment may be indicated in cases resistant to local management or in patients with widespread clinical and dermatological manifestations. Short-term systemic corticosteroid therapy is commonly used in severe acute refractory cases. In adults with severe, treatment-resistant atopic dermatitis, cyclosporine is considered a first-line systemic intervention according to current guidelines [7].

Despite the availability of multiple symptomatic treatment modalities, no therapy can substitute for eliminating allergen exposure. Preventing recurrence also relies heavily on patient education and advice regarding skin barrier protection [7]. This provides patients with a better understanding of how to avoid further exposure and reduce recurrence, rather than relying solely on repeated symptomatic treatment.

Based on the findings of this case and the limited available literature, the authors recommend that patients who require continuous cardiac monitoring and develop suspected electrode-associated contact dermatitis should, whenever feasible, be switched to alternative silver-free electrodes (e.g., radiolucent, dry, or fabric-based) utilizing different adhesive or conductive materials. When continuous monitoring remains clinically necessary, and suitable alternatives are unavailable, a multidisciplinary approach involving dermatology should be considered to minimize cutaneous complications through individualized management strategies, including topical anti-inflammatory therapies, optimization of electrode placement, regular skin assessment, and periodic repositioning of electrodes when clinically appropriate. In cases of recurrent, persistent, or severe reactions, patch testing should be considered to help identify the causative allergen and guide future device selection. Further research is needed to establish evidence-based recommendations for the prevention and management of electrode-associated contact dermatitis in hospitalized patients.

## Conclusions

This case highlights the potential adverse effects of non-invasive interventions routinely used in hospital practice. Physicians should remain aware of the possible consequences of any treatment they initiate, regardless of how common or seemingly harmless it may appear. Additionally, literature regarding the management of complicated cases involving iatrogenic contact dermatitis remains limited, leaving a significant gap in establishing a systematic approach to treatment and optimizing patient care to prevent the development of further serious complications throughout the course of management.

Early dermatology consultation in cases of contact dermatitis can improve patient outcomes in both acute inpatient settings and chronic outpatient care. In this case, the patient’s history of diabetes may have increased the risk of a secondary bacterial infection associated with the rash. A holistic approach to patient care, with prompt recognition and management of adverse reactions, is essential to prevent further complications during hospitalization.
